# Optimization Design and Mechanical Performance Study of Carbon Fiber-Reinforced Composite Load-Carrying Structures for Subway Driver Cabin

**DOI:** 10.3390/ma18112524

**Published:** 2025-05-27

**Authors:** Jinle Wang, Bing Yang, Honglei Tian, Wenbin Wang, Xu Sang

**Affiliations:** 1 State Key Laboratory of Rail Transit Vehicle System, Southwest Jiaotong University, Chengdu 610031, China; yb@swjtu.edu.cn; 2 CRRC Qingdao Sifang Co., Ltd., Qingdao 266111, China; 3 College of Transportation, Tongji University, Shanghai 200092, China; wangwenbin@tongji.edu.cn (W.W.); 2410265@tongji.edu.cn (X.S.)

**Keywords:** subway driver cabin, CFRP, structural optimization, lightweight design, composite layup

## Abstract

This study systematically investigates the optimization design and mechanical performance of carbon fiber-reinforced polymer (CFRP) load-carrying structures for subway driver cabins to meet the lightweight demands of rail transit. Through experimental testing and micromechanical modeling, the mechanical properties of CFRP and foam core materials were characterized, with predicted elastic constants exhibiting an error of ≤5% compared with experimental data. A shape optimization framework integrating mesh morphing and genetic algorithms achieved a 22% mass reduction while preserving structural performance and maintaining load-carrying requirements. Additionally, a stepwise optimization strategy combining free-size, sizing, and stacking sequence optimization was developed to enhance layup efficiency. The final design reduced the total mass by 29.1% compared with the original model, with all failure factors remaining below critical thresholds across three loading cases. The increased failure factor confirmed that the optimized structure effectively exploited the material’s potential while eliminating redundancy. These findings provide valuable theoretical and technical insights into lightweight CFRP applications in rail transit, demonstrating significant improvements in structural efficiency, safety, and manufacturability.

## 1. Introduction

With the rapid development of the rail transit industry and the emergence of new development concepts such as energy conservation, emission reduction, and environmental friendliness, structural lightweighting of rolling stock has become a critical focus in modern vehicle design and manufacturing [1,2]. Carbon fiber-reinforced polymer (CFRP), known for its low weight, high strength, corrosion resistance, cost-effectiveness, and design flexibility in processing and modification, has emerged as a key material for achieving vehicle lightweighting through the manufacture of train structural components [3,4,5].

In contemporary rail vehicle structural design, CFRP applications have progressively expanded from non-load-carrying components to critical load-carrying structures [6,7,8]. European countries, leveraging their advanced composite technology, have implemented CFRP in interior trim elements, energy-absorbing cabin frontends, transition couplers, and pantograph components and have further extended its use to major structures including driver cabins, car bodies, and bogies [9,10,11]. Japan’s N700 series trains adopted CFRP roof structures, effectively lowering the vehicle’s center of gravity while achieving a 500 kg weight-reduction target. Kawasaki Heavy Industries’ efWING bogie, utilizing carbon fiber side beams, demonstrated both lightweight advantages and exceptional safety performance during testing [12]. South Korea’s TTX trains employed carbon fiber–aluminum honeycomb sandwich materials for sidewalls, end walls, and roofs, achieving a 40% overall weight reduction while meeting strength and modal requirements [13,14,15]. Although China initiated CFRP applications in rail transit relatively late, significant breakthroughs have been made in secondary load-carrying structures and components. CRRC Sifang’s 2015-developed CFRP equipment cabin met 350 km/h operational standards with a 35% weight reduction compared with aluminum alloy structures. In 2018, CRRC Changchun pioneered the world’s first full-carbon-fiber metro car body, achieving a 35% weight reduction over conventional metal structures [16,17].

The adoption of lightweight designs for cabin hood or cabin load-carrying structures using higher-strength, lighter-weight CFRP or CFRP–foam sandwich materials represents an effective approach for train lightweighting and a crucial development trend in the future of the rail transit industry. Wang et al. [18] designed the structure of a CFRP train cabin frontend based on the external contour, internal connection requirements, and loading conditions of high-speed trains. They conducted simulation analysis under working conditions specified by relevant standards and performed compression tests on prototypes. The results demonstrated that the mechanical properties of the structure met design requirements. Wang et al. [19] investigated a novel CFRP engine hood, analyzing its strength and deformation under collision, airflow loads, and end-compression conditions. Experimental verification confirmed that the structure satisfied mechanical performance requirements for 500 km/h high-speed test trains. Wang et al. [20] conducted a stepwise optimization design for a subway cabin hood, refining the ply orientation, thickness, and stacking sequence of CFRP panels by integrating manufacturing constraints and engineering experience. Simulation results showed that the optimized composite cabin hood achieved a 27% mass reduction compared with the initial design and a 37% reduction relative to the original glass fiber structure, while meeting strength, stiffness, and stability criteria. Ming et al. [21] developed a self-supporting CFRP subway cabin and systematically investigated the tensile, compressive, flexural, interlaminar shear, and high-velocity impact properties of composite laminates. Their design achieved over 30% weight reduction compared with aluminum alloy cabins. Tang et al. [22] designed CFRP and CFRP–aluminum honeycomb cabin frontends, conducting computational structural analysis to evaluate both configurations’ mechanical performance relative to traditional glass fiber-reinforced plastic (GFRP) structures. All three designs withstood seven working conditions without failure. At equivalent thickness, the CFRP and CFRP-honeycomb configurations achieved 11.18% and 43.80% weight reductions, respectively, versus GFRP, while exhibiting 74.49% and 26.35% increases in initial failure load, with ultimate failure loads improved by 96.45% and 112.06%. Xie et al. [23] combined submodel and structural optimization techniques to enhance CFRP cabin strength in metro vehicles. With ply thickness and orientation as design variables, Tsai–Wu failure index < 0.9 as the constraint, and CFRP mass minimization as the objective, they optimized the cabin frame structure. The final design achieved a failure index of 0.897 and a 33% weight reduction.

Despite advancements in CFRP applications in rail transit, current research on composite cabin frame structures remains limited, particularly in design optimization and systematic investigations of structural failure factor calculations. To address this gap, this study proposes a comprehensive optimization framework for CFRP load-carrying structures in subway driver cabins, integrating multi-stage strategies to enhance both structural efficiency and failure safety. Initially, mechanical property tests were conducted on carbon fiber composites and foam core materials to establish a foundation for subsequent material selection and structural design. The constitutive relationship of continuous carbon fiber composites was predicted using MSC Digimat-MF 2017.0 software based on micromechanics theory, providing fundamental design references for the composite structural design process. For the streamlined driver cabin structure, strength verification of the initial CFRP load-carrying cabin frame was performed under regulatory-specified working conditions. Finite element mesh morphing technology was implemented in ESTECO modeFRONTIER 2019R1 to develop a shape optimization model, wherein a genetic algorithm was utilized for optimizing the geometry of the cabin frame. Furthermore, the principal stress trajectory-based layup design method and the stepwise optimization layup design method were applied to develop an efficient composite layup scheme for the cabin load-carrying structure. The research outcomes provide robust theoretical support and technical references for lightweight design and engineering applications in rail transit equipment.

## 2. Materials and Methods

### 2.1. Material Performance Testing

#### 2.1.1. Mechanical Characterization of CFRP

**Figure 1:**
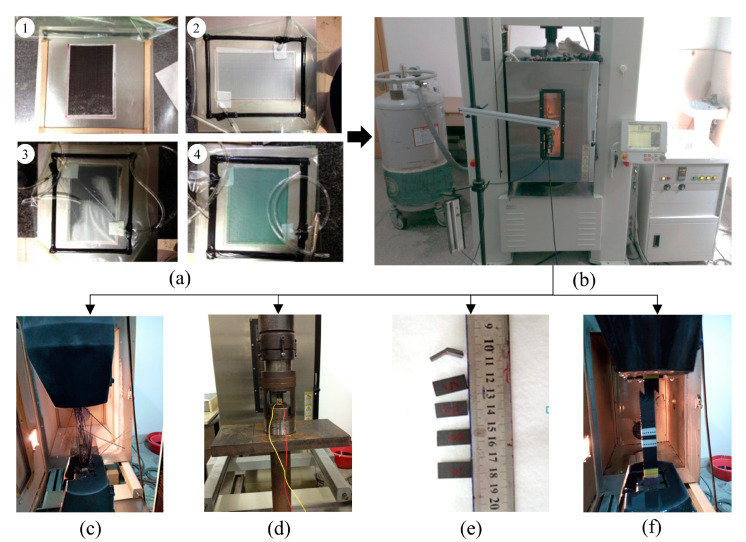
Mechanical tests of T700-12K/Araldite LY1564 SP/Aradur3487 carbon/epoxy system. (**a**) VARI technology: ① fiber fabric lay-up, ② vacuum bagging, ③ resin infusion, ④ cure; (**b**) Shimadzu AG-250kN universal testing machine; (**c**) tensile tests; (**d**) compressive tests; (**e**) interlaminar shear tests; (**f**) in-plane shear tests.

The design of composite components requires fundamental mechanical property data, including constitutive relationships and allowable design values, to determine the material’s composition, fiber content, layup configuration, and structural geometry [24,25]. Unidirectional carbon fiber-reinforced composite laminates were fabricated using Toray T700 fibers (Toray Industries, Inc., Tokyo, Japan) and an Araldite LY1564 SP/Aradur3487 epoxy matrix (Huntsman Corporation, based in The Woodlands, TX, USA). Specimen preparation followed GB4550-2005 [26], employing vacuum-assisted resin infusion (VARI) technology (Figure 1a). Mechanical characterization included tensile, compressive, interlaminar shear, and in-plane shear tests conducted on a Shimadzu AG-250kN universal testing machine at room-temperature conditions (Figure 1b–f). The main material parameters obtained by the tests are shown in Table 1.

#### 2.1.2. Mechanical Characterization of Foam Core Materials

**Figure 2:**
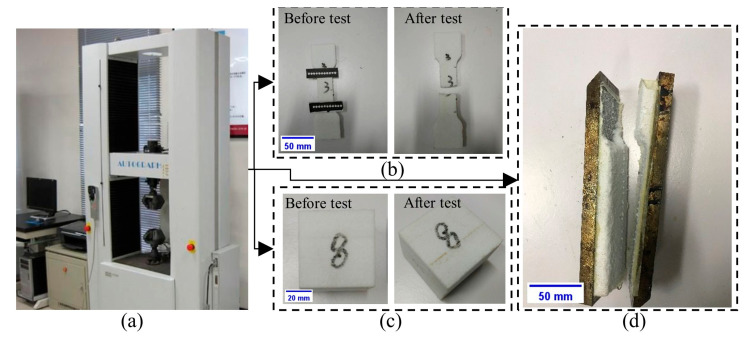
Mechanical tests of ATREX^®^T90 closed-cell thermoplastic structural foam. (**a**) Shimadzu AG-I universal testing machine; (**b**) tensile tests; (**c**) compressive tests; (**d**) shear tests.

Composite sandwich foam cores provided panel separation, structural stabilization, and bending stiffness enhancement while serving as molds in curved configurations [27,28,29]. They primarily resist out-of-plane and transverse shear stresses while contributing to thermal/acoustic insulation—properties fundamentally determined by their mechanical behavior [30]. This study employed ATREX^®^T90 (3A Composites Core Materials, Sins, Aargau, Switzerland) closed-cell thermoplastic structural foam, which is recognized for its superior compressive strength, modulus, fatigue resistance, and creep durability in composite applications. Mechanical characterization included tensile, compressive, and shear tests conducted on a Shimadzu AG-I universal testing machine under room-temperature conditions (Figure 2). The key material parameters obtained by the tests are shown in Table 2.

### 2.2. Constitutive Relation and Strength Theory of Composite Materials

#### 2.2.1. Constitutive Relationship Prediction

Carbon fiber composite constitutive relationships were established through micromechanical modeling using MSC Digimat-MF 2017.0 software. By defining fiber/matrix properties and microstructural parameters, the tool predicted macroscopic elastic constants with ≤5% error relative to experimental data (Table 3), validating its predictive accuracy.

This multi-scale approach enabled efficient composite design by linking microscopic reinforcement configurations to macroscopic properties, eliminating iterative physical testing. The methodology directly interfaced with finite element analysis for cross-scale structural optimization, offering rapid parameter iteration, reduced material input requirements, and seamless integration of micromechanical models with macroscopic engineering workflows.

#### 2.2.2. Material Strength Evaluation Criterion

The Tsai–Wu failure criterion [31,32] was adopted for material strength assessment:

(1) $$I_{F}=\frac{\sigma_{1}^{2}}{X_{T}X_{C}}-\frac{\sigma_{1}\sigma_{2}}{\sqrt{X_{T}X_{C}Y_{T}Y_{C}}}+\frac{\sigma_{2}^{2}}{Y_{T}Y_{C}}+\frac{\tau_{12}^{2}}{S^{2}}+\frac{X_{C}-X_{T}}{X_{T}X_{C}}\sigma_{1}+\frac{Y_{C}-Y_{T}}{Y_{T}Y_{C}}\sigma_{2},$$

where $I_{F}$ is the failure factor when $I_{F}\geq 1$ material failure; $\sigma_{1},\sigma_{2},{\;\tau }_{12}$ are the longitudinal, transverse, and shear stresses of the material, respectively; and $X_{T},{\;X}_{C},{\;Y}_{T},{\;Y}_{C},\;S$ correspond to each strength value in Table 1. This criterion integrates multiple failure modes by incorporating all principal strength parameters, providing a unified characterization of composite material strength.

### 2.3. The Simulation Model

#### 2.3.1. The Subway Cabin Frame Structure

**Figure 3:**
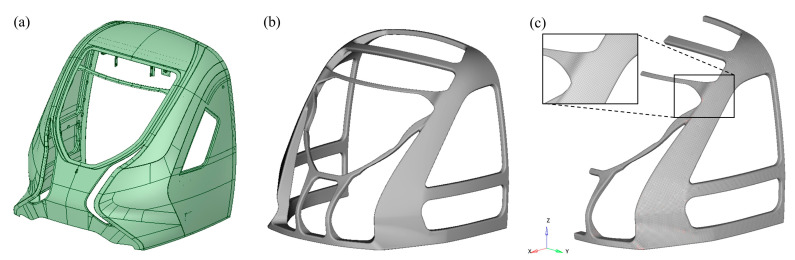
Driver cabin hood model. (**a**) Outer cover; (**b**) load-carrying frame structure; (**c**) finite element model of the load-carrying frame structure.

This study focuses on the carbon fiber structural design of a streamlined cabin frame, using the frame load-carrying structure (Figure 3b) that supports the streamlined outer cover (Figure 3a) as a research prototype. The outer cover transitions smoothly from the headlight holes to the sides and features an inclined windshield. The cabin frame includes primary columns, connecting frames, and crossbeams. The front columns support the outer cover, transfer loads, and provide crash protection. Front brackets and upper crossbeams secure the windshield, while lateral crossbeams reinforce the side windows and provide rigidity. The cabin frame’s exterior maintains curvature matching the enclosure for precise alignment.

**Figure 4:**
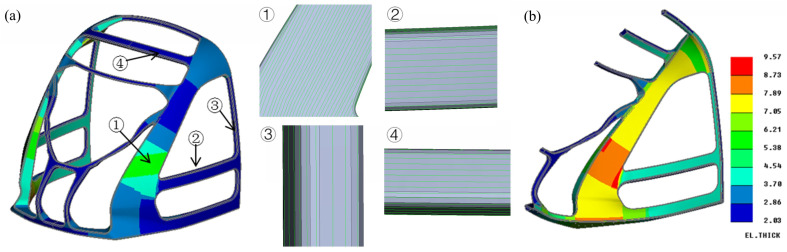
Composite layup design of the cabin frame. (**a**) Local principal direction of composite material: ① front crossbeam, ② inclined column, ③ side crossbeam, ④ rear column; (**b**) thickness distribution of CFRP (mm).

The cabin frame integrated a foam core with surface carbon fiber laminates. This study utilized finite element analysis with Altair OptiStruct 2021 for composite layup design, verification, and optimization. Due to symmetry, a half-model was employed for computational efficiency. The mesh size was set to 15 mm, resulting in a total of 41,739 shell elements for the composite layers and 457,906 solid elements for the foam core, while transitional pyramid and prism elements ensured nodal connectivity between the surface shells and core solids. Material coordinate systems were defined along geometric flow lines to establish layup orientation references, as illustrated in Figure 4a. Initial layup configurations followed an engineering experience-based design incorporating 0°, 90°, +45°, and −45° ply orientations. Critical load-carrying regions, including primary load paths, direct loading zones, and structural corners, received additional ply reinforcement. The thickness distribution mapping in Figure 4b demonstrates the optimized layup strategy, which balances material efficiency with structural performance requirements.

#### 2.3.2. Static Condition

**Figure 5:**
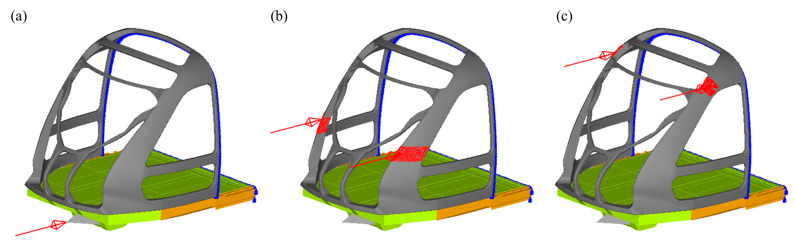
Three computational load cases. (**a**) Case 1: 1200 kN coupler compression; (**b**) Case 2: 300 kN mid-column compression; (**c**) Case 3: 300 kN upper-column compression.

To facilitate load and constraint application, the cabin frame was mounted on the vehicle body underframe. Considering computational scale, especially during optimization iterations where a single cycle’s load impacts total time, only a portion of the underframe was used as the cabin frame’s mounting base in the early design stage. The static strength evaluation of the CFRP cabin frame adhered to EN12663-1:2010 standards [33], focusing on three critical load cases to establish baseline performance for structural optimization. Case 1 applied a 1200 kN longitudinal compressive load at the coupler mounting point (Figure 5a). Cases 2 and 3 imposed 300 kN distributed loads at the lower and upper regions of the front columns, respectively, with the forces equally divided between the left and right sides through nodal force distribution (Figure 5b,c). In all load cases, full constraints were implemented at all nodes along the rear cabin frame and simplified underframe interfaces, with rigid connections established between the cabin base and underframe to replicate actual assembly conditions.

## 3. Results

### 3.1. Static Strength Analysis of Initial Structure

**Figure 6:**
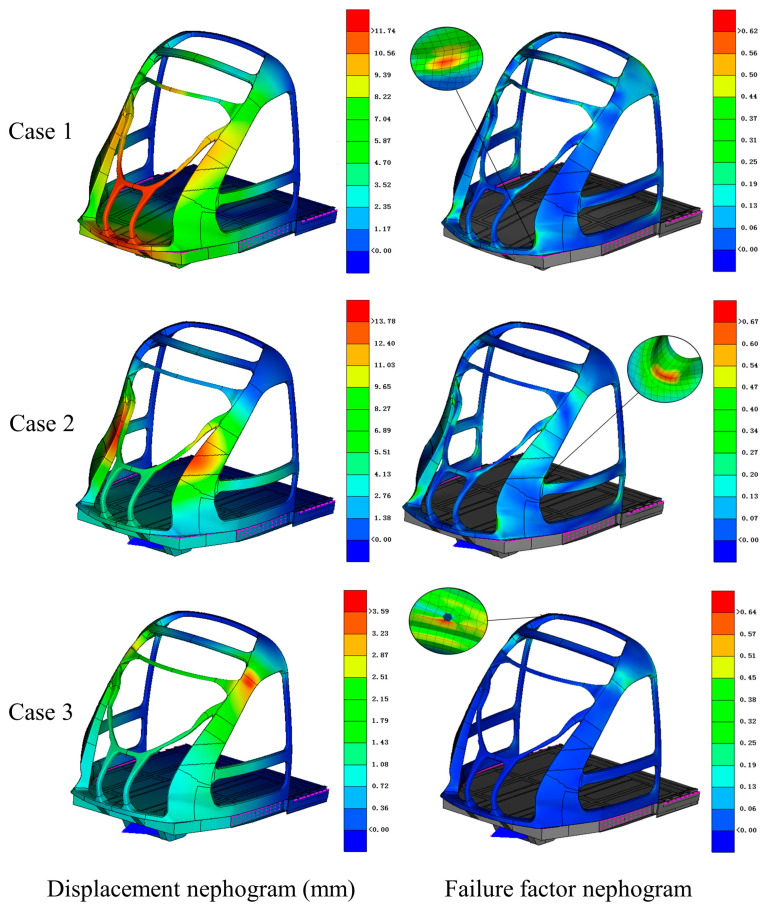
Displacement and failure factor cloud diagrams of the initial structure under three load cases.

Three critical load cases were systematically analyzed. As shown in Figure 6, under Case 1, with a 1200 kN coupler compression load, downward bending deformation propagated from the coupler to the front bracket via the base connection, resulting in a maximum displacement of 11.74 mm. Stress concentration at the primary column–base interface reached a failure factor of 0.62. Case 2, involving 300 kN mid-column compression, induced a three-point bending mode, causing 13.78 mm displacement and a peak failure factor of 0.67 at the column–side beam transition. Case 3, with 300 kN upper-column loading, exhibited minimal deformation (3.59 mm) due to force alignment with the laminate plane, although a failure factor of 0.64 was observed near the bolt holes. The failure factors in all three load cases remained in a subcritical state, confirming the structural integrity while highlighting the key areas for optimization.

### 3.2. Cabin Frame Shape Optimization

**Figure 7:**
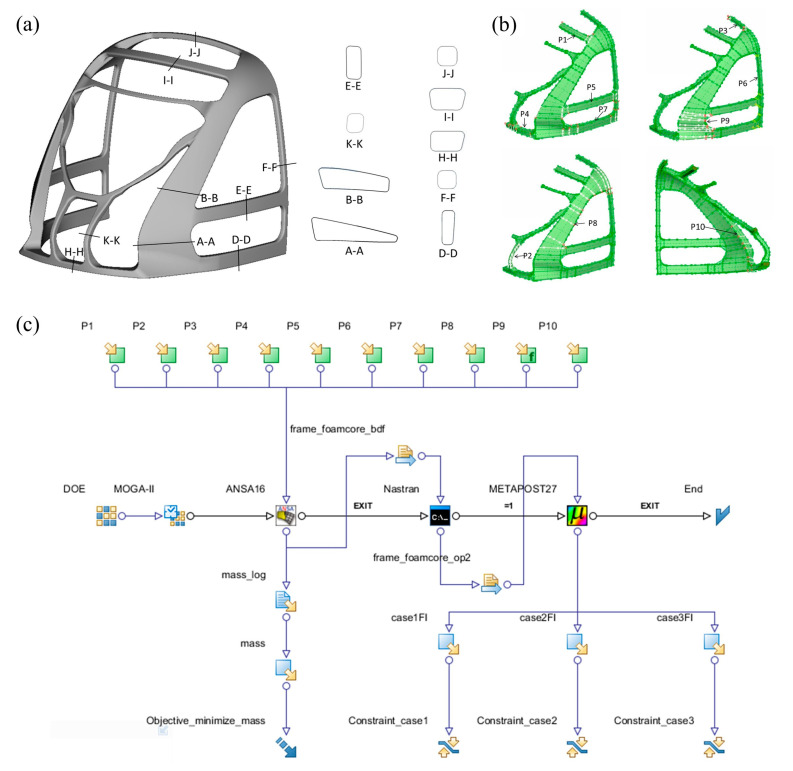
(**a**) Schematic diagram of the cross-section of the driver cabin frame; (**b**) the selection of design variables in shape optimization; (**c**) the workflow of shape optimization in modeFRONTIER.

The shape optimization of the cabin frame concentrated on modifying the cross-sectional profiles of the columns and beams to enhance strength, stiffness, and mass efficiency while maintaining interface integrity with external components (Figure 7a). To improve computational efficiency, a symmetric half-model was utilized. ANSA’s Box-Morphing technique was applied to encapsulate the cabin frame using hexahedral and prismatic control boxes. Three parametric deformation modes governed the optimization process: (1) boundary sliding of control nodes (e.g., parameters P3–P5, P7–P10), (2) normal offsetting of box faces to adjust column widths (e.g., P2), and (3) longitudinal translation along the X-axis (e.g., P6). The design variables controlled the magnitudes of these deformations (Figure 7b). The optimization model was established by using the optimization software ESTECO modeFRONTIER 2019R1, integrating design variables, optimization algorithms, logical control, mesh deformation, computational solving, and finite element result post-processing into an optimization workflow that includes logical control flow and data flow, as shown in Figure 7c. The optimized mathematical model constructed is shown in Equation (2).

(2) $$\left\{\begin{array}{l} find:P={\left[P_{1},P_{2},\ldots ,P_{10}\right]}^{T}\\ min:W(P)=W\\ s.t.:g_{j}(P)-g_{j}^{U}\leq 0,\;j=1,2,3\\ \;\;\;\;\;\;\;\;P_{k}^{L}\leq P_{k}\leq P_{k}^{U},\;i=1,2\ldots ,10 \end{array}\right.$$

where P is a vector composed of 10 deformation parameters extracted based on mesh morphing technology; W represents the total mass of the structure; $g_{j}\left(P\right)$ denotes the three strength constraint conditions corresponding to the load cases for frame strength calculation established in Section 2.3.2; $g_{j}^{U}$ is the upper limit for the j-th load case, where all failure factors must remain below 1; $P_{k}^{L}$ and $P_{k}^{U}$ are the lower and upper limits of the k-th design variable, respectively.

**Figure 8:**
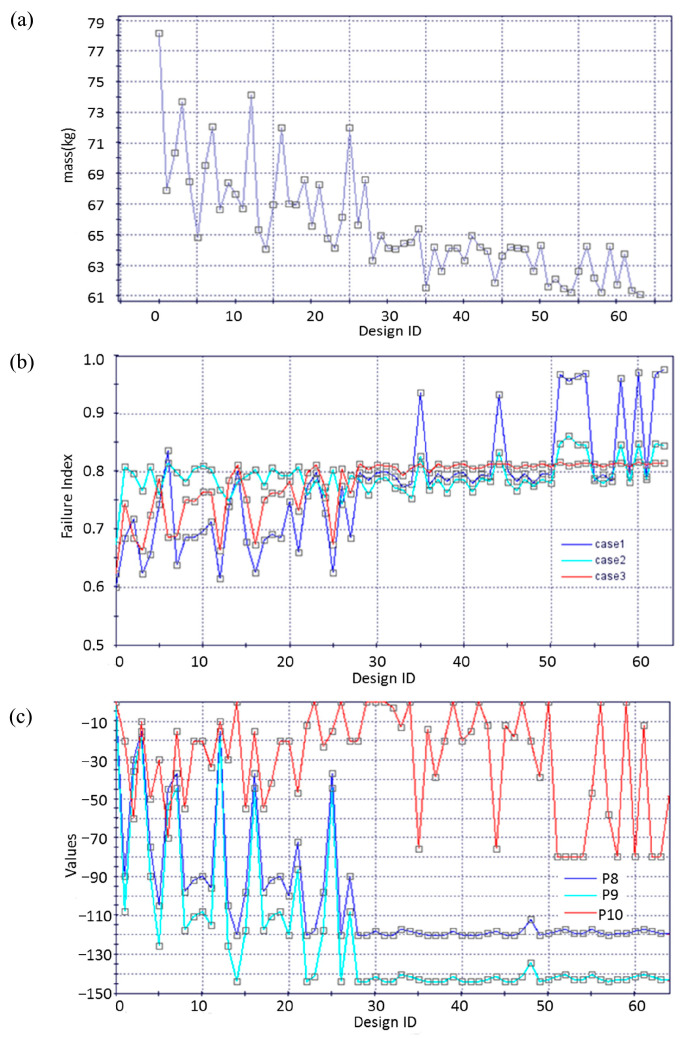
The evolution of (**a**) objective functions (total mass of the cabin frame), (**b**) constraint responses (failure factors), and (**c**) key design variables during optimization.

Figure 8 shows the evolution of objective functions, constraint responses, and key design variables during optimization. It highlights discrete, non-sequential adjustments in design variables typical of genetic algorithms that involve stochastic selection, crossover, and mutation guided by fitness evaluations. As shown in Figure 8c, variables such as P8 and P10 stabilize once their associated fitness reaches the optimum. In contrast, variables like P10, which are sensitive to the objective function and constraint conditions, continue to undergo genetic variation trials during the later stages of optimization. This is because P10 strongly correlates with the failure factor response in Case 1, where changes in P10 significantly affect the distance between the inner and outer surfaces of the main column. In bending scenarios, placing material farther from the neutral axis improves structural efficiency.

As shown in Figure 8a,b, after 63 generations of genetic algorithm optimization, the total mass of the 1/2 model of the cabin frame was reduced from 78.15 kg in the initial design to 61.12 kg, achieving a 22% weight reduction. The maximum stress failure factors for the three strength verification cases improved from the original values of 0.60, 0.67, and 0.62 to 0.98, 0.85, and 0.81, respectively, all remaining below 1 and within the allowable safety range. Using the mesh morphing approach, the shape optimization of the cabin frame effectively achieved lightweight design while ensuring functional performance and material strength safety.

### 3.3. Laminate Design Based on Stepwise Optimization

**Figure 9:**
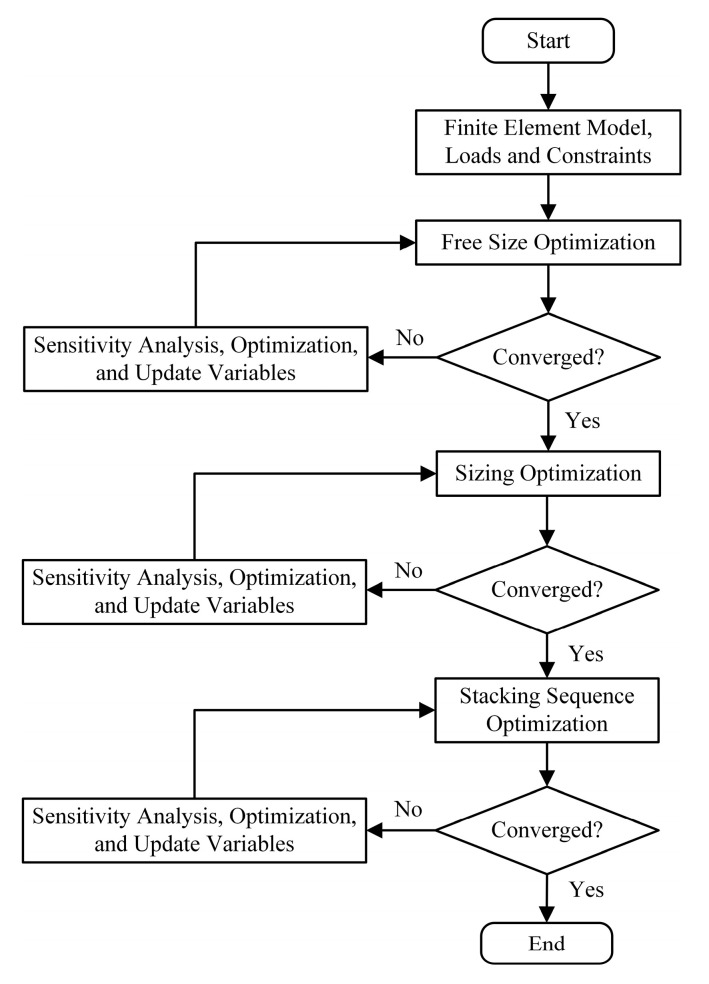
Flowchart of stepwise optimization design for composite materials.

The structures of composite materials are complex and varied, and their optimization design involves a multitude of variables, including both continuous and discrete variables [34,35]. Furthermore, the intercoupling among these variables significantly increases the complexity and difficulty of the optimization process. This paper employs a stepwise optimization strategy using OptiStruct. Design variables are categorized based on their characteristics and optimized sequentially in groups. The complex composite material optimization problem is divided into three stages: free-size optimization (conceptual design), size optimization (system design), and stacking sequence optimization (detailed design). In each stage, finite element analysis is used to evaluate structural responses, followed by convergence checks and sensitivity analyses to develop approximate models. Subsequently, the physical model is transformed into a mathematical one, and optimal solutions are obtained under multiple constraints using optimization theory and mathematical programming methods. The stepwise optimization process for composite materials is illustrated in Figure 9.

#### 3.3.1. Free-Size Optimization

Free-size optimization establishes the global material distribution for each ply orientation in the cabin frame’s inner and outer panels, emphasizing fiber alignment along load transfer paths to enhance axial stiffness and strength while addressing shear and bending requirements [36,37]. Four primary orientations—0°, 90°, and ±45°—are strategically utilized: 0° plies improve axial load-carrying capacity, ±45° plies reduce shear stresses and enhance manufacturability, and 90° plies control transverse stiffness and Poisson’s ratio. By grouping same-direction plies into super layers, this method simplifies the computational complexity and accelerates convergence by neglecting stacking sequence effects.

Design variables include ply thickness per element, with the goal of minimizing structural compliance under combined loading conditions. Constraints ensure strength, stiffness, and buckling stability across three load cases as well as manufacturability considerations: the upper limit of the volume fraction of the composite material is 0.3, a minimum of 20% and maximum of 70% thickness per orientation, symmetrical ±45° ply configurations to avoid torsional stresses. The optimized mathematical model constructed is shown in Equation (3).

(3) $$\left\{\begin{array}{l} find:X_{i}=(X_{1},X_{2}\cdots ,X_{NE})\\ min:f(X_{i})=C\\ s.t.:g_{j}(X)-g_{j}^{U}\leq 0,\;j=1,2,3\\ \;\;\;\;\;\;\;\;manufacturability \end{array}\right.$$

where $X_{i}$ represents the thickness of plies in each orientation for element i, with NE being the total number of elements; C denotes the structural compliance; $g_{j}\left(X\right)$ and $g_{j}^{U}$ are the response and upper limit for the j-th load case, respectively. Three load cases are included.

**Figure 10:**
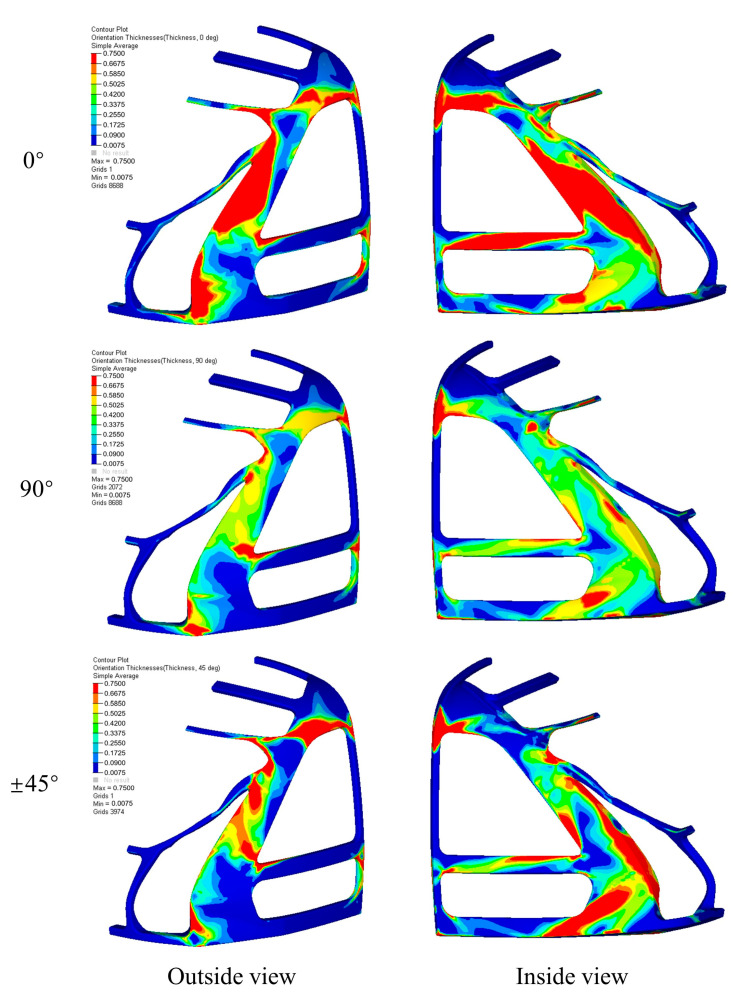
The thickness distribution of each ply of the inner and outer panels after free size optimization.

The initial design of the layup orientations for the inner and outer panels of the driver cabin frame is 45°, 0°, −45°, and 90°. Each ply has an initial thickness of 0.75 mm, and the total thickness is 3 mm. Figure 10 illustrates the thickness distribution of each ply for the inner and outer panels after free-size optimization, defining the shapes of the ply blocks in the super layers at various angles. Each super layer at a specific angle consists of four plies, with varying shapes and thicknesses, stacked to ensure continuous thickness transitions and prevent abrupt changes in ply block thickness.

#### 3.3.2. Sizing Optimization

The continuous thickness variations obtained from free-size optimization are not directly manufacturable [38]. Practical designs necessitate ply thicknesses as integer multiples of the minimum single-ply thickness (0.15 mm). In this phase, the free-size results are refined by removing isolated ply regions, merging small blocks, and smoothing boundaries to improve producibility. Design variables primarily involve ply-block thicknesses, with the goal of minimizing total structural mass. Constraints enforce discrete thickness increments (0.15 mm multiples) alongside prior manufacturing rules. The optimized mathematical model constructed is shown in Equation (4).

(4) $$\left\{\begin{array}{l} find:t_{l}=(T_{1},T_{2},\cdots ,T_{n})\\ min:W(t_{i})=W\\ s.t.:g_{j}(X)-g_{j}^{U}\leq 0,\;j=1,2,3\\ \;\;\;\;\;\;\;\;manufacturability \end{array}\right.$$

where $t_{l}$ is the thickness of the l-th ply layer, with $T_{l}$ being discrete thickness values determined by manufacturing constraints, and W is the total structural mass.

**Figure 11:**
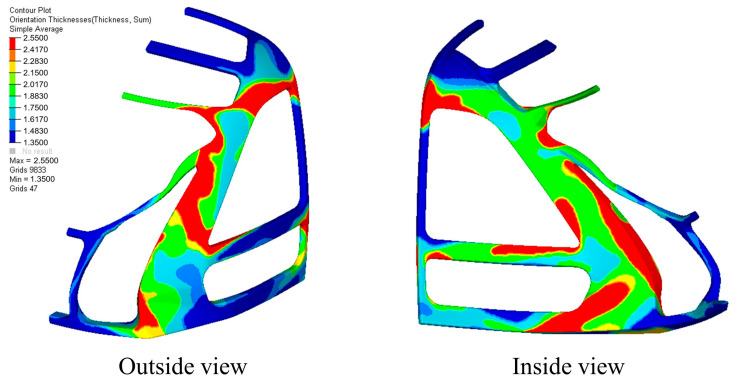
The thickness distribution of each ply of the inner and outer panels after sizing optimization.

Figure 11 illustrates the total thickness distribution of the driver cabin frame structure after size optimization. The structure comprises 6 layers at 0°, 5 layers at 90°, and 3 layers each at ±45°, forming a total of 17 ply blocks with varying shapes. These blocks are stacked without considering the influence of ply sequence. Each ply block has a uniform thickness of 0.15 mm. The thickest region of the structure measures 2.55 mm and is located in the red area of the figure, while the thinnest region measures 1.35 mm and is located in the dark blue area.

#### 3.3.3. Stacking Sequence Optimization

Stacking sequence optimization plays a crucial role in balancing mechanical performance and manufacturability for composite structures [39,40,41]. In this phase, laminate stiffness is maximized by optimizing the arrangement of plies based on the sizing optimization results, ensuring an even distribution of ply orientations throughout the thickness to avoid localized weaknesses. Constraints restrict consecutive plies of the same orientation to no more than four layers, thereby reducing the risk of interlaminar stresses. The optimized mathematical model constructed is shown in Equation (5).

(5) $$\left\{\begin{array}{l} find:\;\varphi_{m}={\left[\varphi_{\sigma 1},\varphi_{\sigma 2},\cdots ,\varphi_{\sigma N}\right]}^{T}\\ min:f\left(\varphi_{m}\right)=C\\ s.t.:g_{j}(X)-g_{j}^{U}\leq 0,\;j=1,2,3\\ \;\;\;\;\;\;\;\;manufacturability \end{array}\right.$$

where $\varphi_{m}$ is the angle vector composed of laminate blocks $\varphi_{\sigma 1},\varphi_{\sigma 2},\cdots ,\varphi_{\sigma N}$, with σ denoting permutation operators; C is the structural compliance.

**Figure 12:**
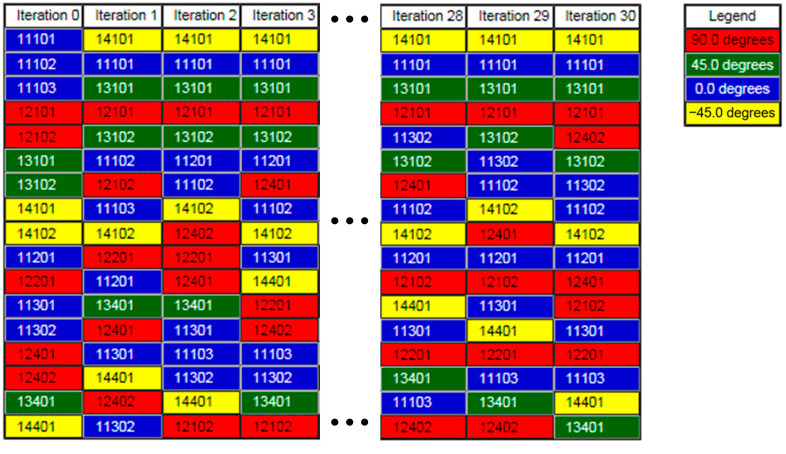
The process of optimizing the stacking sequence.

After 30 iterations, the optimized stacking sequences (Figure 12) demonstrate global ply layouts that are specifically tailored to meet load requirements. As a result of the shape trimming of each ply during free-size optimization, most plies fail to fully cover the cabin frame load-carrying structure. This leads to ply transition zones being exposed on the outermost layer of the laminate. To prevent delamination failure at these step-like boundaries, a continuous covering ply must be applied on the surface. In order to enhance the maintainability of the ply structure and simultaneously improve the laminate’s compressive and impact resistance, ±45° full-size plies are incorporated on the surface of the optimized stacking sequence.

After obtaining the optimization results, a finite element model of the driver cabin frame load-carrying structure was developed, and failure factor analysis was performed under three static loading cases. The optimized results were compared with the pre-optimization analysis data and the original layup mode (see Table 4 for details). Through a three-step optimization process combined with composite material layup engineering experience, the final 1/2 symmetrical model of the driver cabin frame achieved a mass of 55.41 kg, representing a 16.4% reduction compared with the pre-optimization model. The strength failure factors were significantly reduced. Compared with the original layup model presented in Section 3.1, the mass was decreased by 29.1%, and although the strength failure factor increased, all values remained below 1, ensuring the structure is within the safe limits of material integrity. The increased failure factor of the optimized structure demonstrates that by aligning the composite material layup structure and material property layout with the load conditions, the full potential of the materials was utilized, effectively eliminating structural strength redundancy.

## 4. Conclusions

This study proposes a novel multi-stage optimization framework combining micromechanical modeling, shape optimization via mesh morphing, and stepwise layup design to advance lightweight CFRP structures in rail vehicles, using a subway train driver cabin frame as a case study. The key findings are summarized as follows:

(1) The Digimat-MF platform, based on composite micromechanics, demonstrates effective prediction of the constitutive relationships of continuous carbon fiber composites. Inputting fiber/matrix constitutive relationships and microstructure parameters, the predicted macroscopic elastic constants of unidirectional laminates show maximum absolute errors below 5% (peak value 4.7%) compared with experimental data. Under the simulated loading conditions, the mechanical response exhibits substantial consistency with that observed under actual test loading conditions within the elastic limit.

(2) Mesh morphing technology enables direct geometric modification of finite element meshes while maintaining layup reference coordinates and mesh quality for composite structures. Integrated with a shape optimization model, this approach successfully optimizes the geometric configuration of driver cabin frame structures. Using genetic algorithms, the optimized half-model achieves 22% mass reduction, from 78.15 kg to 61.12 kg, while maintaining smooth geometric transitions suitable for direct application. The modular optimization framework demonstrates clear logic, operational efficiency, and excellent scalability for diverse engineering applications.

(3) The stepwise optimization methodology progressively determines fiber orientations, ply thicknesses, and stacking sequences through free-size optimization, size optimization, and stacking sequence optimization. By integrating composite engineering experience, the final optimized half-model achieves a mass of 55.41 kg (a 16.4% reduction compared with the pre-optimization model) with significantly improved strength failure factors. Compared with the original design, this represents a 29.1% mass reduction while ensuring all failure factors remain below 1.0, thereby guaranteeing structural integrity. The increased failure factor confirms that the optimized material layout and layup configuration effectively exploit material potential while eliminating structural redundancy.

These innovations advance the field of composite structural design by balancing computational efficiency, manufacturability, and performance in rail applications. It should be noted that the current work focuses solely on the static load conditions specified by EN12663 standards [33], and future studies could extend this framework to dynamic or fatigue loading scenarios for comprehensive validation.

## Figures and Tables

**Table 1:** Mechanical properties of T700-12K/Araldite LY1564 SP/Aradur3487 carbon/epoxy system.

Properties	Value
Elastic properties	$E_{1}=128.69\pm 2.30\;GPa$, $E_{1c}=144.85\pm 16.45\;GPa$, $E_{2}=8.97\pm 0.42\;GPa$, $E_{2c}=12.28\pm 1.73\;GPa$, $G_{12}=3.76\pm 0.09\;GPa$
Material strengths	$X_{T}=2463.24\pm 92.37\;MPa$, $X_{C}=748.77\pm 63.65\;MPa$, $Y_{T}=44.42\pm 3.30\;MPa$, $Y_{c}=126.26\pm 10.83\;MPa$, $S_{12}=70.10\pm 0.86\;MPa$, $S_{23}=62.95\pm 1.69\;MPa$

Note: $E_{1}$ represents longitudinal tensile modulus, $E_{1c}$ represents longitudinal compressive modulus, $E_{2}$ represents transverse tensile modulus, $E_{2c}$ represents transverse compressive modulus, and $G_{12}$ represents shear modulus; $X_{T}$ represents longitudinal tensile strength, $X_{C}$ represents longitudinal compressive strength, $Y_{T}$ represents transverse tensile strength, $Y_{c}$ represents transverse compressive strength, $S_{12}$ represents shear strength, and $S_{23}$ represents interlaminar shear strength.

**Table 2:** Mechanical properties of ATREX^®^T90 closed-cell thermoplastic structural foam.

Properties	Value
Elastic properties	$E_{T}=65.36\pm 3.78\;MPa$$,\;E_{C}=72.99\pm 4.35\;MPa$, G=37.08±1.39 MPa
Material strengths	$\sigma_{b}=1.85\pm 0.04\;MPa$$,\;\sigma_{bc}=2.04\pm 0.05\;MPa$, S=1.37±0.04 MPa

Note: $E_{T}$ represents tensile modulus of foam core materials, $E_{C}$ represents compressive modulus, and G represents shear modulus; $\sigma_{b}$ represents tensile strength, $\sigma_{bc}$ represents compressive strength, and S represents shear strength.

**Table 3:** Comparison of the predicted and experimental values of macroscopic elastic constants of carbon fiber unidirectional composites.

Properties	Predicted Value (GPa)	Experimental Value (GPa)	Error (%)
$E_{1}$	134.68	128.69	4.7
$E_{2}$	8.69	8.97	−3.1
$G_{12}$	3.93	3.76	4.5

**Table 4:** Performance comparison of the composite material driver cabin frame load-carrying structure before and after optimization.

Layup Design	Failure Factor			Total Mass(kg)
	Case 1	Case 2	Case 3	
Original layup model	0.62	0.67	0.64	78.15
Pre-optimization model	0.78	0.92	0.59	66.28
Stepwise optimization model	0.73	0.72	0.57	55.41

## Data Availability

The original contributions presented in this study are included in the article. Further inquiries can be directed to the corresponding author.
